# Emotions as Abstract Evaluation Criteria in Biological and Artificial Intelligences

**DOI:** 10.3389/fncom.2021.726247

**Published:** 2021-12-14

**Authors:** Claudius Gros

**Affiliations:** Institute for Theoretical Physics, Goethe University Frankfurt am Main, Frankfurt, Germany

**Keywords:** emotion theory, feelings (emotions), theory mind, artificial intelligence, decision making

## Abstract

Biological as well as advanced artificial intelligences (AIs) need to decide which goals to pursue. We review nature's solution to the time allocation problem, which is based on a continuously readjusted categorical weighting mechanism we experience introspectively as emotions. One observes phylogenetically that the available number of emotional states increases hand in hand with the cognitive capabilities of animals and that raising levels of intelligence entail ever larger sets of behavioral options. Our ability to experience a multitude of potentially conflicting feelings is in this view not a leftover of a more primitive heritage, but a generic mechanism for attributing values to behavioral options that can not be specified at birth. In this view, emotions are essential for understanding the mind. For concreteness, we propose and discuss a framework which mimics emotions on a functional level. Based on time allocation via emotional stationarity (TAES), emotions are implemented as abstract criteria, such as satisfaction, challenge and boredom, which serve to evaluate activities that have been carried out. The resulting timeline of experienced emotions is compared with the “character” of the agent, which is defined in terms of a preferred distribution of emotional states. The long-term goal of the agent, to align experience with character, is achieved by optimizing the frequency for selecting individual tasks. Upon optimization, the statistics of emotion experience becomes stationary.

## 1. Introduction

Humans draw their motivations from short- and long term objectives evolving continuously with new experiences (Huang and Bargh, 2014). Here we argue that this strategy is dictated in particular by the fact that the amount of information an agent disposes about the present and the future state of the world is severely constraint, given that forecasting is intrinsically limited by successively stronger complexity barriers (Gros, 2012). Corresponding limitations hold for the time available for decision making and for the computational power of the supporting hard- or wetware (Zenon et al., 2019; Lieder and Griffiths, 2020), independently of whether the acting agent is synthetic or biological. A corollary of this observation is that the time allocation problem, which goals to pursue consecutively, cannot be solved by brute force computation. Instead, nature disposed us with an emotional control system. It is argued, in consequence, that an improved understanding of the functional role of emotions is essential for theories of the mind.

Emotions have emerged in the last decades as indispensable preconditions for higher cognition (Panksepp, 2004; Gros, 2010). It has been pointed out, in particular, that the core task of an emotional response is not direct causation of the type “fleeing because one is afraid,” but the induction of cognitive feedback, anticipation, and reflection (Baumeister et al., 2007). Being afraid will in general not result in a direct behavioral reaction, but in the allocation of cognitive resources to the danger at hand. If there is chance, it is better to attempt to solve an existing problem. It has been shown in this context that emotional and cognitive processes form a tight feedback system in terms of emotional priming of cognition (Beeler et al., 2014), and cognitive control of emotions (Ochsner and Gross, 2005). Cognitive emotion regulation (Inzlicht et al., 2015), such as the attempt to restrain one's desire for unhealthy food, is present to such a stage (Cutuli, 2014), that it can be regarded as a defining characteristics of our species. The advanced cognitive capabilities that are paramount to efficiently pursue a given goal, like winning a game of Go, will hence leave their imprints also on the cogno-emotional feedback loop (Miller and Clark, 2018).

On a neuronal level it has been observed (Pessoa, 2008) that the classical characterization of brain regions as “affective” and “cognitive” is misleading (Pessoa, 2019). The reason is that complex cogno-emotional behaviors are based rather on dynamic coalitions of networks of brain areas (Pessoa, 2018), than on the specific activation of a single structure, such as the amygdala (Phelps, 2006). The same holds for the neural representations of the cognitive control mechanisms regulating emotional responses, which are found to be distributed within a network of lateral frontal, temporal, and parietal regions (Morawetz et al., 2016).

The interconnection of cognitive and emotional brain states suggests a corresponding dual basis for decision making (Lerner et al., 2015). Logical reasoning would then be responsible to analyze alternative choices, with the outcome of the different choices being encoded affectively as values (Reimann and Bechara, 2010). An equivalent statement holds for the weighting of the associated risks (Panno et al., 2013), in particular when it comes to long-term, viz strategic decision taking (Gilkey et al., 2010). One feels good if a specific outlook is positive and certain, and uneasy otherwise. The consequence is hence that intelligence is needed to rationalize decision options, but that intelligence alone, if existing in terms of a pure logical apparatus, cannot solve the time allocation problem. Logic is not enough to decide which long-term goals to pursue one after another. It has been argued, in analogy, that self-control is intrinsically not a purified rational, but a value-based decision process (Berkman et al., 2017).

The picture emerging is that the brain uses deductive and other types of reasoning for the analysis of behavioral options, see (Shepard and Metzler, 1971; Johnson-Laird, 2010; Papo, 2015), and emotional states for the weighting of the consequences. One observes that distinct types and combination of emotional states, like anger, envy, trust, satisfaction, etc, are attributed to specific types of behavioral options (Pfister and Böhm, 2008; Schlösser et al., 2013), which implies that the number of emotional states an agent needs for the weighting of its options increases hand in hand with the complexity of decision making. A consequence of this interrelation between emotional and cognitive complexity shows up in daily life, to give an example, when it comes to handle adversity effectively, which has been shown to benefit from emotional diversity (Grossmann et al., 2019).

As an intriguing corollary of here discussed scenario we note that an hypothetical artificial intelligence of human and transhuman level should dispose, as a conditio sine qua non, of a palette of emotional states containing ever finer shades of states. Synthetic emotions would be in this framework equivalent to human emotions on a functional level, but not necessarily in terms of corresponding qualia. The reason for the increased emotional sensibility of advanced AIs is that the number of available weighting categories has to match the increased number of behavioral and cognitive options accompanying high intellectual capabilities. The challenge to guarantee the long term dynamical stability of the corresponding feedback loop between motivations, goal selection and introspective cognitive analysis, viz the task to control advanced AIs, will hence increase in complexity with raising intelligence levels.

It has been observed that one obtains an improved level of understanding if working principles for the brain are not only formulated, but also implemented algorithmically (Cauwenberghs, 2013; Hawkins, 2017). In this spirit we will present, after discussing the relation between emotions and feelings, a simple but operational cogno-emotional framework. The goal is to show that the concept of emotional stationarity allows to select varying timelines of subsequent tasks. Cognitive abilities are in this view important to complete a given task, with emotional values being responsible for task selection in first place.

For our discussion, a multi-gaming environment will be used as a reference application. Within this protocol, agents have two qualitatively different tasks. First to select what to do, the time allocation problem at its core, and then to complete the selected job. Having finished a game, say Go or chess, agents will evaluate the acquired experience emotionally, with the timeline of experiences shaping in turn the decision process of what to do next.

### 1.1. Life-Long Utility Maximization

From the perspective of Darwinian evolution, life-long utility maximization is directly proportional to the number of offsprings. For most humans, this is nowadays not the goal, if it ever has been. As mentioned before, we presume here that the aim is instead to align character and experiences and that this process is mediated by emotions. Support from neuropsychological research will be discussed further below. As a matter of principle one could imagine, alternatively, that life-long utility maximization is achieved by calculating at any moment the optimal course of action, while discounting the entirety of future rewards. For a variety of reasons this is however not possible, even if exceedingly large computational powers would be at one's disposal.

In machine learning, the scaling of performance with the amount of dedicated resources has been investigated. Within the domain of language processing, it has been shown that the performance of deep-learning architectures scales as a power-law of any of the three primary scarce resources, time, model- and training-data size (Kaplan et al., 2020), if not bottlenecked by one of the others. It is good news that exponentially larger amounts of resources are not needed to boost the performance within a well specified application domain. The computational demands needed by top deep architectures increases in contrast exponentially with time (Agneeswaran, 2020), at a rate that outpasses Moore's law by far (Geifman, 2020). From the perspective of complex systems theory (Gros, 2015), this is not a surprise, given that state-of-the-art machine learning architectures are applied to increasingly complex problems and domains. For example, when forecasting horizons are extended, the intrinsically chaotic nature of most complex systems demands exponentially increasing computing times. This difficulty has been termed “complexity barrier” (Gros, 2012).

In societies, complexity barriers arise in addition from the need to predict and to interpret the behavior of the other members, which is of course a reciprocal task. Indeed, the “social brain hypothesis” (Dunbar, 2009) states that a core evolutionary driver for the development of our brains has been the need to deal with the complexity of human social systems, with the latter evolving in parallel with increasing brain sizes. From an evolutionary perspective, a cognitive intra-species arms race with progressively increasing computational resources leads to a “red-queen phenomenon” Dieckmann et al. (1995), namely that it takes “all the running to stay in place.” These two factors, the eventual occurrence of an intra-societal cognitive arms race, and the intrinsic complexity of the environment, makes it impossible to predict the future via brute force computations, in particular for the purpose to maximize life-long utilities. It is to be seen if an analogous line of arguments holds for societies of advanced artificial intelligences.

## 2. Experiencing Emotions As Feelings

Before delving further into the analysis of the functional role of emotions, we take a step back and ask a deceivingly simple question. Why do we have feelings in first place?

At any given point of time, a myriad of neural, chemical and electrical processes take place in our brains. For the overwhelming part, consciously we are however not aware of what our supporting wetware is doing (Van Gaal and Lamme, 2012; Dehaene et al., 2014). In contrast, we are able to experience as feelings (Wang and Pereira, 2016) the class of processes corresponding to emotional states (Colibazzi et al., 2010). Why then has evolution developed neural circuits allowing our brain to experience feelings? The alternative would be that the functional role of emotions would be performed by neural processes we could not register consciously. In this case we would be akin to what has been called at times a “zombie” (Koch and Crick, 2001), viz a human-like being which is not aware of its emotional drives (Winkielman and Berridge, 2004). A zombie would just go for the food, when hungry, without being able to restrain itself. Defined as such, zombies are not able to close the cogno-emotional loop (Inzlicht et al., 2015; Miller and Clark, 2018), lacking the capability to control emotions cognitively. The human condition is based, in contrast, on emotional control as a defining trait. This is the underlying reason why people with reduced impulse control skills, e.g., when intoxicated or drunk, are considered more often than not to be less accountable for their doings (Penney, 2012), at times to the extent that they are exempted from criminal liability.

It is presently not fully settled how we are able to experience the feelings arising in conjunction to emotional brain states. A series of experiments point in this regard to a feedback loop involving the response of the body (Levenson, 2014). The prospect would be that emotional brain states invoke bodily reactions, like an increased heart-beating rate, that would be transmitted back as “gut feelings” to the brain via propriosensation (Nummenmaa et al., 2014), that is through visceral and other peripheral sensors (Kreibig, 2010). Of interest is here that the cortical region responsible for channeling the afferent propriosensation, the anterior insular cortex, is fully developed only in higher apes and hence phylogenetically young (Craig and Craig, 2009). Animals unable of self recognition seem to lack the spindle-shaped economo neurons characteristic of the anterior insular cortex. Deactivating the brain regions allowing us to sense our own body would bring us hence one step closer to losing the ability to experience emotional states as feelings (De Sousa, 2010). Given that evolution has taken care to equip us with feelings, they must improve Darwinian fitness, entailing hence important functionalities.

A vast number of studies has shown that emotional processes regulate the attributing of values to stimuli (Cardinal et al., 2002) and that they bind conceptual information through affective meaning (Roy et al., 2012). Being able to experience these brain processes consciously in terms of feelings is therefore a necessary condition for the conscious control of the brain's value system. Feelings are in this view the keystone closing the feedback circle between cognitive information processing and the emotional value system. Our preferences and disinclinations would be fully subconscious, and not controllable, if we would not be able to perceive them introspectively as feelings. This line of arguments, which relates the introspective experience of emotional states to the ability to be aware of one's own value system, is in our view likely to be the rationale for the phylogenetic emergence of feelings.

### 2.1. Emotions in Non-human Animals

Emotions are not unique to humans (LeDoux, 2012), but functional states of the nervous system that can be studied across species (Anderson and Adolphs, 2014; Perry and Baciadonna, 2017). An emerging consensus in the field is that animal and human emotions have functional equivalent roles with regard to decision making (Mendl and Paul, 2020). Going down the phylogenetic tree, the decreasing complexity of the nervous system entails however that the range of possible affective states narrows progressively. For example, it has been observed that fish may appraise environmental stimuli cognitively (Cerqueira et al., 2017), that flies can express anxiety (Mohammad et al., 2016), and that the decision-making behavior in bumblebees seems analogous to optimism in humans (Perry et al., 2016), at least on an operational level (Baracchi et al., 2017). It is however difficult to imagine that a fly could take pride in her doings, or experience any other of the myriads of human emotional state that obtain their significance from social context.

Humans are set apart from the other animals populating earth not only because of their cognitive capabilities, but also because of their ability to experience not just a few, but a vast variety of emotional states. Studies of heartbeat perception tasks have found, as discussed in the previous section, that the substrate for subjective feeling states is provided by a phylogenetically young brain region, the anterior insular cortex (Craig and Craig, 2009), via a representation of evoked visceral responses (Critchley et al., 2004). The anterior insular cortex plays however not only a prominent role in the experience of emotions, but also in the value attribution system, enabling behavioral flexibility (Ebitz and Hayden, 2016; Kolling et al., 2016). During decision-making, the dorsal anterior cingulate cortex is thought to regulate the tradeoff between exploring alternative choices, and persistence. A related viewpoint links the dorsal anterior cingulate cortex to the allocation of computational resources to decision making (Shenhav et al., 2016). From a somewhat philosophical point of view one may hence ask whether it is a coincidence, a caprice of nature (Gros, 2009), to say, that humans are at the same time the most intellectual and the most emotional species (Mendl et al., 2011; Maximino et al., 2015). It may alternatively be a conditio sine qua non. Higher cognitive powers would be in the latter case dependent on an equally evolved emotional system (Vitay and Hamker, 2011).

## 3. An Exemplary Cogno-Emotional Framework

In the following we provide an example for a bare-bone cogno-emotional architecture. The aim is to demonstrate that our proposed concept, emotions as abstract evaluation criteria, is valid in the sense that it can be implemented algorithmically. No claims are made that the framework examined, TAES (“time allocation via emotional stationarity”), has direct correspondences to specific brain states or processes. For illustrational purposes, an application scenario from machine learning is used (Rumbell et al., 2012; Jordan and Mitchell, 2015). Specifically, we discuss a multi-gaming environment, viz the case that the agent, f.i. a machine-learing AI, decides on its own which game to play next.

### 3.1. Multi-Gaming Environments

Modern machine-learning algorithms based on deep-neural nets are able to play a large variety of distinct games (Schrittwieser et al., 2020), such as Go, chess and Starcraft, or console games like Atari. We consider a setup where the opponents may be either human players that are drawn from a standard internet-based matchmaking system, standalone competing algorithms, or agents participating in a multi-agent challenge setup (Samvelyan et al., 2019). Of minor relevance to the question at hand is the expertise level of the architecture and whether game-specific algorithms are used. A single generic algorithm (Silver, 2017), such as standard Monte Carlo tree search supplemented by a value and policy generating deep network (Silver et al., 2017), would do the job. For our purpose, the key issue is not the algorithm actually playing, but the question whether the process determining which task to select, viz which game to play at any given time, is universal. In particular, we demand that the task selection process can be adapted in a straightforward manner when the palette of options is enlarged, f.i. when the possibility to connect to a chat room is added.

We stress that the framework introduced here, TAES, is rudimentary on several levels. A fully developed cogno-emotional feedback loop is not present, which is in part because present-day agents are neither able to reflect on theirselves, no to reason rationally on a basic level. TAES serves however as an implementable illustration of emotional task selection and evaluation.

### 3.2. Emotional Evaluation Criteria

In a first step one needs to define the qualia of the emotional states and how they are evaluated, viz the relation of distinct emotions to experiences. The following definitions serve as examples.

– *Satisfaction.* Winning a game raises the satisfaction level. This could hold in particular for complex games, that is for games that are characterized, f.i., by an elevated diversity of game situations.
– *Challenge.* Certain game statistics may characterize a game as challenging. An example would be games for which the probability to win dropped temporarily precariously low.
– *Boredom.* Games for which the probability to win remains constantly high could be classified as boring or, alternatively, as relaxing. The same holds for overly long games.

**Figure 1:**
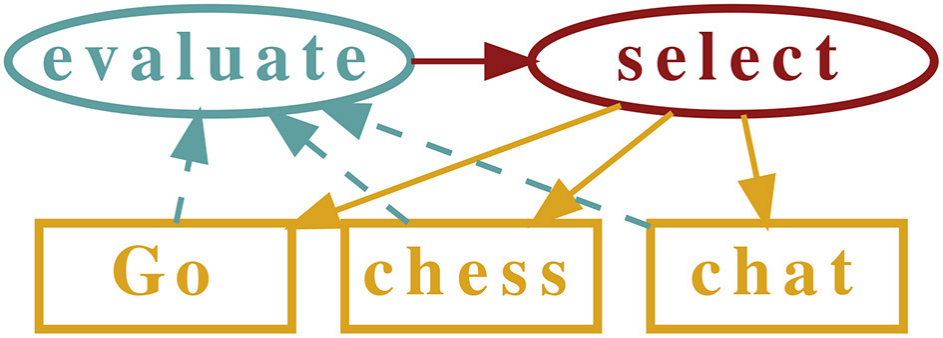
Illustration of a general time-allocation framework. The different options, here to play Go, to play chess and to chat, are evaluated emotionally once completed, with the evaluation results feeding back into the decision what to do next. TAES, time allocation via emotional stationarity, is a specification of the general framework.

The key point of above examples is that they can be implemented algorithmically. Once a task is performed, which means that the game is played till the end, the history of moves can be analyzed and the game classified algorithmically along above emotional criteria. See Figure 1 for an illustration.

The implementation of the evaluation procedure depends on the computational framework used. Consider f.i. the generic deep architectures AlphaGo (Silver et al., 2017) and AlphaZero (Silver et al., 2018), which consist of layered networks with two heads, one for the policy and one for the value, together with a Monte-Carlo tree search for valuable game positions. For a given game position, the value head outputs an estimate for the probability to win. A game could be classified hence as boring when the chance to win, as predicted by the value head, remains constantly high, say above 70%. The policy head suggests likewise possible high yielding moves, which helps to guide the generation of the Monte Carlo search tree. A tree characterized by a single main stem proposes only a limited number of possible good moves. A complex and widely branched tree would in contrast be equivalent to a challenging situation, with larger numbers of possible moves. An elevated frequency of complex search trees would classify a game therefore as challenging. These two examples of evaluation criteria abstract from the semantic content of what the agents is actually doing, a defining property. They are hence suitable to evaluate if any full-information two-player game without random components, the application domain of AlphaGo and AlphaZero, is boring or challenging.

Emotions correspond to value-encoding variables, denoted for above example with *S*, *C* and *B*, respectively for satisfaction, challenge and boredom. Alternative emotional qualia would be defined equivalently. It is important note to keep in mind that the aim of our framework is to model the core functionality of human emotions, but not necessarily their affective meanings, which implies that it is not mandatory for the evaluation criteria to resemble human emotions in terms of their respective qualia. The latter is however likely to make it easier, f.i., to develop an intuitive understanding of emotionally-driven robotic behavior.

### 3.3. Direct Emotional Drivings vs. Emotional Priming

Standard approaches to modeling synthetic approaches often assume that emotional state variables are explicit drivers of actions (Rodŕıguez and Ramos, 2015), either directly or via a set of internal motivations (Velsquez, 1997). This means that a state variable corresponding f.i. to being 'angry' would be activated by specific events, with the type of triggering stimuli being hard coded, viz specified explicitly by the programmer. Here we are interested in contrast in frameworks that are generic, in the sense that behavior is only indirectly influenced by emotional states (Beeler et al., 2014). This implies, as illustrated in the previous section, that emotional evaluation abstracts in its basic functionality from semantic content.

Within TAES, the agent updates in a first step its experience. For every type of activity, say when playing Go, the probability that a game of this type is challenging, boring or satisfying is continuously updated. It could turn out, e.g., that Go games are typically more challenging and less boring than chess games. Based on this set of data, the experience, the next game will be selected with the aim to align experience as close as possible with the “character” of the agent, which is defined in the following.

### 3.4. Aligning Experience With Character

We define the character **C**_*A*_ of the agent as a preset probability distribution of emotional states,


(1) $$\begin{array}{l} {\text{C}}_{A}={\{P}_{S},P_{C},P_{B}\},\;P_{S}+P_{C}+P_{B}=1, \end{array}$$

where *P*_*S*_, *P*_*C*_, *P*_*B*_ ≥ 0 are the target frequencies to experience a given emotional state. The character is hence defined as the set of individual preferences. Agents with large *P*_*C*_ / *P*_*B*_ would prefer for example challenging / boring situations. The overall objective function of the agent is to align experience with its character. This means that agent aims to experience satisfying, challenging and boring situations on the average with probabilities *P*_*S*_, *P*_*C*_ and *P*_*B*_.

**Figure 2:**
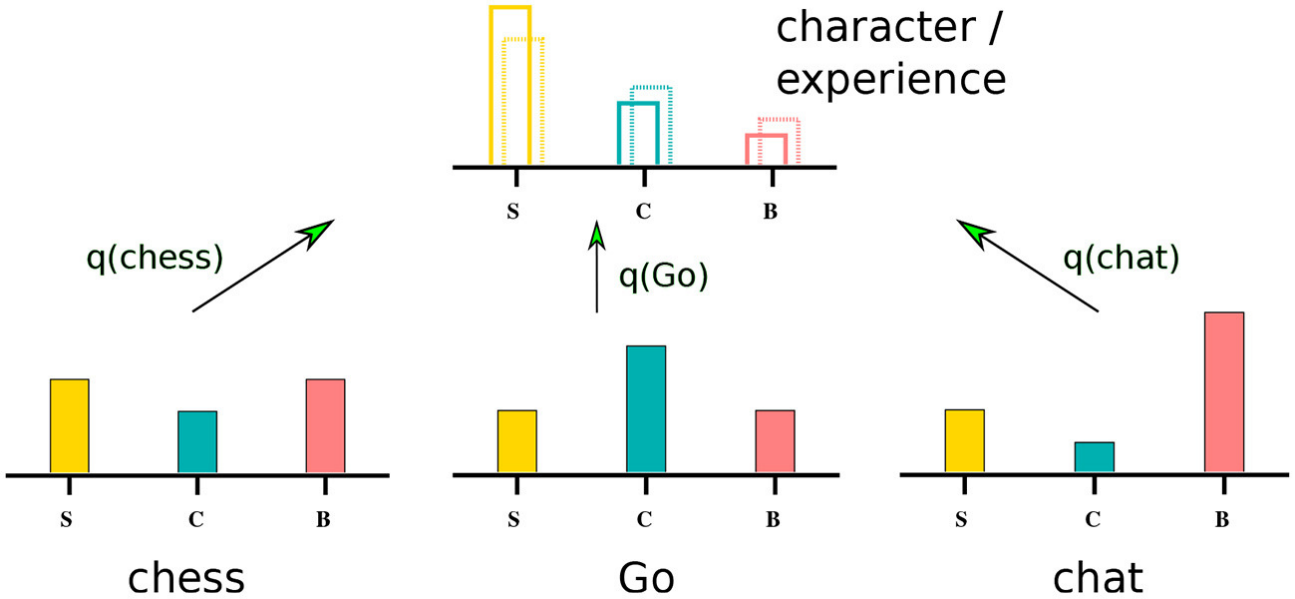
Aligning experience with character. Behavioral options (playing chess, playing Go, joining a chat) are evaluated along emotional criteria, such as being satisfying (S), challenging (C) or boring (B). The corresponding probability distributions are superimposed with weights *q*_α_ = *q*(α), where α ∈ {chess, Go, chat}. See Equation (3). The goal is to align a predefined target distribution of emotional states, the character, with the actual emotional experience. This can be achieved by optimizing the probabilities *q*_α_ to engage in activity α.

On a basic level, experience can be expressed as a set of *N* probability distribution functions,


(2) $$\begin{array}{l} {\text{E}}^{\alpha }={\{p}_{S}^{\alpha },p_{C}^{\alpha },p_{B}^{\alpha }\},\;\alpha =1,\ldots ,N, \end{array}$$

where *N* is the number of possible activities (playing Go, chess, connecting to a chat room,...). For every option α the agent records, as illustrated in Figure 2, the probability $p_{i}^{\alpha }$ for the activity to be satisfying/challenging/boring (*i* = *S*/*C*/*B*). Defining with *q*_α_ the likelihood to engage in activity α, the overall experience **E**_*A*_ is given as


(3) $$\begin{array}{l} {\text{E}}_{A}=\underset{\alpha }{\sum }q_{\alpha }{\text{E}}^{\alpha },\;\underset{\alpha }{\sum }q_{\alpha }=1, \end{array}$$

where the **E**^α^ are defined in Equation (2). The global objective, to align character **C**_*A*_ and experience **E**_*A*_, can be achieved by minimizing the Kullback-Leibler divergence between **C**_*A*_ and **E**_*A*_ with respect to the *q*_α_. This strategy, which corresponds to a greedy approach, could be supplemented by an explorative component allowing to sample new opportunities (Auer, 2002). Modulo exploration, an activity α is hence selected with probability *q*_α_.

TAES is based on aligning two probability distribution functions, **E**_*A*_ and **C**_*A*_, an information-theoretical postulate that has been denoted the “stationarity principle” (Echeveste et al., 2015) in the context of neuronal learning (Trapp et al., 2018) and critical brain activity (Gros, 2021). It states that not the activity as such should be optimized, but the distribution of activities. The resulting state is consequently varying in time, but stationary with respect to its statistical properties. The underlying principle of the here presented framework corresponds to “time allocation via emotional stationarity” (TAES). Within this approach, the character of the agent serves as a guiding functional, a stochastic implementation of the principle of guided self-organization (Gros, 2014).

### 3.5. Motivational Drives

Up to now we considered purely stochastic decision making, namely that activities are selected probabilistically, as determined by the selection probabilities *q*_α_. An interesting extension are deterministic components corresponding to emotional drives. Considering finite time spans, we denote with *p*_*i*_(*N*_*a*_) the relative number of times that emotion *i* = *S, C, B*, ... has been experienced over the course of the last *N*_*a*_ activities. Ideally, the trailing averages *p*_*i*_(*N*_*a*_) converge to the desired frequencies *P*_*i*_, see Equation (1). Substantial fluctuations may however occur, for example when the agent is matched repeatedly to opponents with low levels of expertise, which may lead to an extended streak of boring games. The resulting temporary discrepancy,


(4) $$\begin{array}{l} M_{i}=P_{i}-p_{i}\left(N_{a}\right), \end{array}$$

between desired and trailing emotion probabilities can then be regarded as an emotional drive. Stochastically, *M*_*i*_ averages out for appropriate probabilities *q*_α_ to select an activity α. On a shorter time scales one may endorse the agent with the option to reduce excessive values of *M*_*k*_ by direct action, viz by selecting an activity β = Go, Chess, ... characterized by large/small $p_{k}^{\beta }$ when *M*_*k*_ is strongly positive/negative. This is meaningful in particular if the distribution $\left\{p_{i}^{\beta }\right\}$ is peaked and not flat. Emotional drives correspond to an additional route for reaching the overall goal, the alignment of experience with character.

### 3.6. Including Utility Maximization

In addition to having emotional motivations, agents may want to maximize one or more classical reward functions, like gaining credits for wining games, or answering a substantial number of questions in a chat room. Without emotional constraints, the program would just select the most advantageous option, once the available options have been explored in sufficient depth for their properties, in analogy to the multi-armed bandit problem (Vermorel and Mohri, 2005). An interesting constellation arises when rewards are weighted emotionally, e.g., with the help of the Kullback-Leibler divergence *D*_α_ between the character and the emotional experience of a given behavioral option (Gros, 2015),


(5) $$\begin{array}{l} D_{\alpha }=\underset{i}{\sum }P_{i}\log\left(\frac{P_{i}}{p_{i}^{\alpha }}\right). \end{array}$$

Credits received from a behavioral option α that conforms with the character of the agent, having a small *D*_α_, would be given a higher weight than credits gained when engaging in activities characterized by a large *D*_α_. There are then two conflicting goals, to maximize the weighted utility and to align experience with character, for which a suitable prioritization or Pareto optimality may be established (Sener and Koltun, 2018).

Instead of treating utility as a separate feature, one may introduce a new emotional trait, the desire to receive rewards, viz to make money, and subsume utility under emotional optimization on an equal footing. Depending on the target frequency *P*_*U*_ to generate utility, the agent will select its actions such that the full emotional spectrum is taken into account. A separate weighting of utility gains, as expressed by (5), is then not necessary.

## 4. Discussion

Computational models of emotions have focused traditionally on the interconnection between emotional stimuli, synthetic emotions and emotional responses (Rodŕıguez and Ramos, 2015). A typical goal is to generate believable behavior of autonomous social agents (Scherer, 2009), in particular in connection with psychological theories of emotions, involving f.i. appraisal, dimensional aspects or hierarchical structures (Rodŕıguez and Ramos, 2015). Closer to the scope of the present investigation are proposals relating emotions to learning and with this to behavioral choices (Gadanho, 2003). One possibility is to use homeostatic state variables, encoding f.i. “well-being,” for the regulation of reinforcement learning (Moerland et al., 2018). Other state variables could be derived from utility optimization, like water and energy uptake, or appraisal concepts (Moerland et al., 2018), with the latter being examples for the abstract evaluation criteria used in the TAES framework. One route to measure well-being consist in grounding it on the relation between short- and long-term trailing reward rates (Broekens et al., 2007). Well-being can then be used to modulate dynamically the balance between exploitation (when doing well) and exploration (when things are not as they used to be). Alternatively, emotional states may impact the policy (Kuremoto et al., 2013).

Going beyond the main trust of research in synthetic emotions, to facilitate human-computer interaction and and to use emotions to improve the performance of machine learning algorithms that are applied to dynamic landscapes, the question that has been asked here regards how an ever ongoing sequence of distinct tasks can be generated by optimizing emotional experience, in addition to reward. Formulated as a time allocation problem, the rational of our approach is drawn mainly from affective neuroscience (Gros, 2009), and only to a lesser extent from psychological conceptualizations of human emotional responses. Within this setting, the TAES framework captures the notion that a central role of emotions is to serve as abstract evaluation tools that are to be optimized as a set, and not individually. This premise does not rule out alternative emotional functionalities.

Emotions are considered to be grounded in “affect,” viz in the brain states mediating pleasant and unpleasant arousal (Wilson-Mendenhall et al., 2013). This seems at first a contradiction to the notion that emotions correspond to “abstract” evaluation criteria, as advocated here. It is worthwhile to point out in this context that emotions are intrinsically related to “domain-general” neural processes (Barrett, 2009; Chen et al., 2018), and that moral judgments seem to recruit, on a related note, domain-general valuation mechanisms on the basis of probabilistic representations (Shenhav and Greene, 2010). One can be frustrated when failing to perform while playing violin, to illustrate this point, or when getting a ticket for driving too fast. Frustration may arise, like any other emotional state, in highly diverse domains. In this sense, domain-general processes and valuation mechanisms can be termed to be abstract.

### 4.1. Testing of Functional Emotional Frameworks

For living beings, capabilities are selected ultimately when they contribute to evolutionary success. This holds in particular also for emotional regulation. A closely related area is the formation of moral preferences, an issue that is examined by a rapidly growing body of game-theoretical approaches (Capraro and Perc, 2021). Engaging in seemingly unselfish behavior comes in this view with personal benefits. In this context, rational choice theory presumes that agents act rationally, given their personal resource limitations and preferences (Dietrich and List, 2013).

Classical game-theoretical concepts can be tested using suitable laboratory protocols (Camerer and Ho, 2015). Evidence becomes somewhat more indirect when the direct maximization of monetary utility is complemented by personal preferences that are hypothesized to include moral components, like fairness and retaliation (Fehr and Gächter, 2000). Testing conceptual frameworks for game-theoretical settings in which moral preferences are allowed to evolve is even less straightforward (Chandan, 2016). This observation holds also for the here proposed framework, TAES, in which preferences, f.i. to engage in challenging tasks, may fluctuate strongly, being defined only by their long-term average. Any protocol for testing emotional frameworks will be bounded by this caveat.

Detailed testing protocols for TAES are yet to be developed. They would be based in any case on a setting, in which participants are given not one, but several different tasks to perform. It would be up to the participants to select the relative frequency, viz the number times they engage in any one of the possible tasks. The individual tasks would be conceptually similar, differing however quantitatively along several feature dimensions. For example one task could be complex, but mildly challenging, another seemingly simple, but somewhat difficult. In order to include variability, one could include a simple but strongly varying type of task. The timeline of task selection would then be compared with a previously taken character evaluation of the participant. The outcome of the experiment would be in agreement with TAES if character and the statistics of the timeline of actions would align.

## 5. Conclusions

The here developed concept, time allocation via emotional stationarity (TAES), can be seen from two viewpoints. On one side as a guiding hypothesis for studies of the brain. TAES serves in this context as an example for the working of emotions in terms of abstract evaluation criteria. On the other side, TAES can be seen as a first step toward the implementation of truely synthetic emotions, viz emotions that mirror human emotion not only on a phenomenological, but on a functional level.

Frameworks for synthetic emotions are especially powerful and functionally close to human emotions if they can be extended with ease along two directions. Firstly, as argued in this study, when the protocol for the inclusion of new behavioral options is applicable to a wide range of activity classes. This is the case when emotions do not correspond to specific features, but to domain-general evaluation criteria. Essentially any type of activity can then be evaluated, f.i., as being boring, challenging, risky, demanding, easy, and so on. It is also desirable that the framework allows for the straightforward inclusion of new traits of emotions, such as longing for monetary rewards.

Frameworks for the understanding of the emotional system should be able to explain that humans dispose of characteristic personalities (DeYoung and Gray, 2009; McNaughton and Smillie, 2018). For theories of emotions this implies that there should exist a restricted set of parameters controlling the balancing of emotional states in terms of a preferred distribution, the functional equivalent of character. As realized by the TAES framework, the overarching objective is consequently to adjust the relative frequencies to engage in a specific task, such that the statistics of the experienced emotional states aligns with the character.

Human life is characterized by behavioral options, such as to study, to visit a friend, or to take a swim in a pool, that have strongly varying properties and multi-variate reward dimensions. It is hence questionable whether utility optimization in terms of a univariate money-like credit, e.g., as for the multi-armed bandit problem, would suffice for an understanding of human motivational drives. A resolution of this conundrum is the concept of emotions as domain-general evaluation criteria. In this perspective, life-long success depends not only on the algorithmic capability to handle specific tasks, but also on the alignment of experiences and personality.

## Data Availability Statement

The original contributions presented in the study are included in the article/supplementary material, further inquiries can be directed to the corresponding author/s.

## Author Contributions

The author confirms being the sole contributor of this work and has approved it for publication.

## Conflict of Interest

The author declares that the research was conducted in the absence of any commercial or financial relationships that could be construed as a potential conflict of interest.

## Publisher's Note

All claims expressed in this article are solely those of the authors and do not necessarily represent those of their affiliated organizations, or those of the publisher, the editors and the reviewers. Any product that may be evaluated in this article, or claim that may be made by its manufacturer, is not guaranteed or endorsed by the publisher.
